# BEDB: a comprehensive binding energy database for molecular docking and dynamics: insights into Human Metapneumovirus (HMPV) Inhibitors

**DOI:** 10.1093/database/baag011

**Published:** 2026-02-27

**Authors:** Farhan Ullah, Wajeeha Rahman, Anees Ullah, Riffat Jehan, Ali Raza, Shamsher Khan, Zarmeena, Shahid Ullah, Tianshun Gao

**Affiliations:** Lab for Computational and Structural Biology, College of Life Science and Technology, Huazhong University of Science and Technology, 1037# Luoyu Road, Wuhan, Hubei, 430074, China; School of Materials Science and Engineering, Harbin Institute of Technology, Shenzhen 518055, China; S Khan Lab Mardan, Khyber Pakhtunkhwa, Pakistan; School of Materials Science and Engineering, Harbin Institute of Technology, Shenzhen 518055, China; S Khan Lab Mardan, Khyber Pakhtunkhwa, Pakistan; S Khan Lab Mardan, Khyber Pakhtunkhwa, Pakistan; S Khan Lab Mardan, Khyber Pakhtunkhwa, Pakistan; S Khan Lab Mardan, Khyber Pakhtunkhwa, Pakistan; Clinical Big Data Research Center, Shenzhen Key Laboratory of Bone Tissue Repair and Translational Research, Department of Orthopaedic Surgery, The Seventh Affiliated Hospital of Sun Yat-Sen University, Shenzhen, Guangdong 518107, P.R. China

## Abstract

Biological databases play a crucial role in life sciences research by organizing vast amounts of data, enabling efficient access and analysis. Numerous databases have been published across various research areas, yet there remains a need for updated platforms in the field of molecular docking and molecular dynamics simulation research. To address this gap, we have developed an extensive and user-friendly platform focused on compiling the binding energies of compounds associated with a wide range of biological activities. The database offers free access to data on 1321 compounds, including abstracts, references, isomeric SMILES, and 22 molecular properties. Researchers can also securely store their docking and screening data. To demonstrate its capabilities, molecular docking was performed on the top 10 compounds with the best binding energies against human metapneumovirus (HMPV) using AutoDock Vina and the crystal structure (PDB ID: 8FPJ). MK-3207 and Etoposide exhibited docking scores of −10.3 and −9.6, respectively. The top two compounds were further selected for MD simulations, confirming stable binding interactions with the viral protein. Additional compounds, including Teniposide, UK432097, 85019940, Setileuton, Orvepitan, Cep-11981, Tadalafil, and VS-5584, were also analyzed, providing further insights into their binding mechanisms and potential therapeutic relevance. The database is developed using PHP, HTML, CSS, JavaScript, and Python and is freely accessible at https://www.pbed.habdsk.org/.

## Introduction

Biological databases are essential tools in the life sciences, facilitating the organization, access, and analysis of extensive biomedical data [1–12]. Simultaneously, docking and simulation databases play a vital role in structure-based drug design by storing computational models of molecular interactions, docking scores, and simulation data [13–15]. These repositories enable rapid screening of compound libraries and predict binding affinities between ligands and target proteins, thereby accelerating the identification of potential drug candidates [16–19].

In recent years, the proliferation of various biological databases has significantly transformed research methodologies. However, a pressing need remains for updated platforms, especially in the areas of molecular docking and molecular dynamics (MDs) simulations. With the rapid advancement of computational technologies and an increasing interest in natural products as therapeutic agents, developing a dedicated database that addresses these needs is crucial for fostering innovation in drug discovery. To bridge this gap, we have developed a comprehensive and user-friendly platform focused on compiling binding energy data for compounds associated with diverse biological activities. Our database currently includes information on 1321 compounds, encompassing detailed abstracts, references, isomeric SMILES, and 22 molecular properties. In addition to being a valuable repository of information, the platform allows researchers to securely store their molecular docking and screening data, thereby enhancing the reproducibility and transparency of scientific research.

In demonstrating the capabilities of our database, we conducted molecular docking studies on the top 10 compounds with the highest binding energies against human metapneumovirus (HMPV). Previously, significant work has been conducted on Mpro inhibitors using docking and MDs, including the identification of antiviral phytochemicals and the computational investigation of natural compounds for their efficacy [12, 19,20]. The analysis revealed that the top two compounds, MK-3207 and etoposide, exhibited strong binding interactions with the viral protein, suggesting their potential as antiviral agents. The database has been built using a combination of PHP, HTML, CSS, JavaScript, and Python. This integrated approach not only enhances the research landscape but also paves the way for new therapeutic solutions derived from different compounds.

## Material and method

### Construction of BEDB

**Figure 1:**
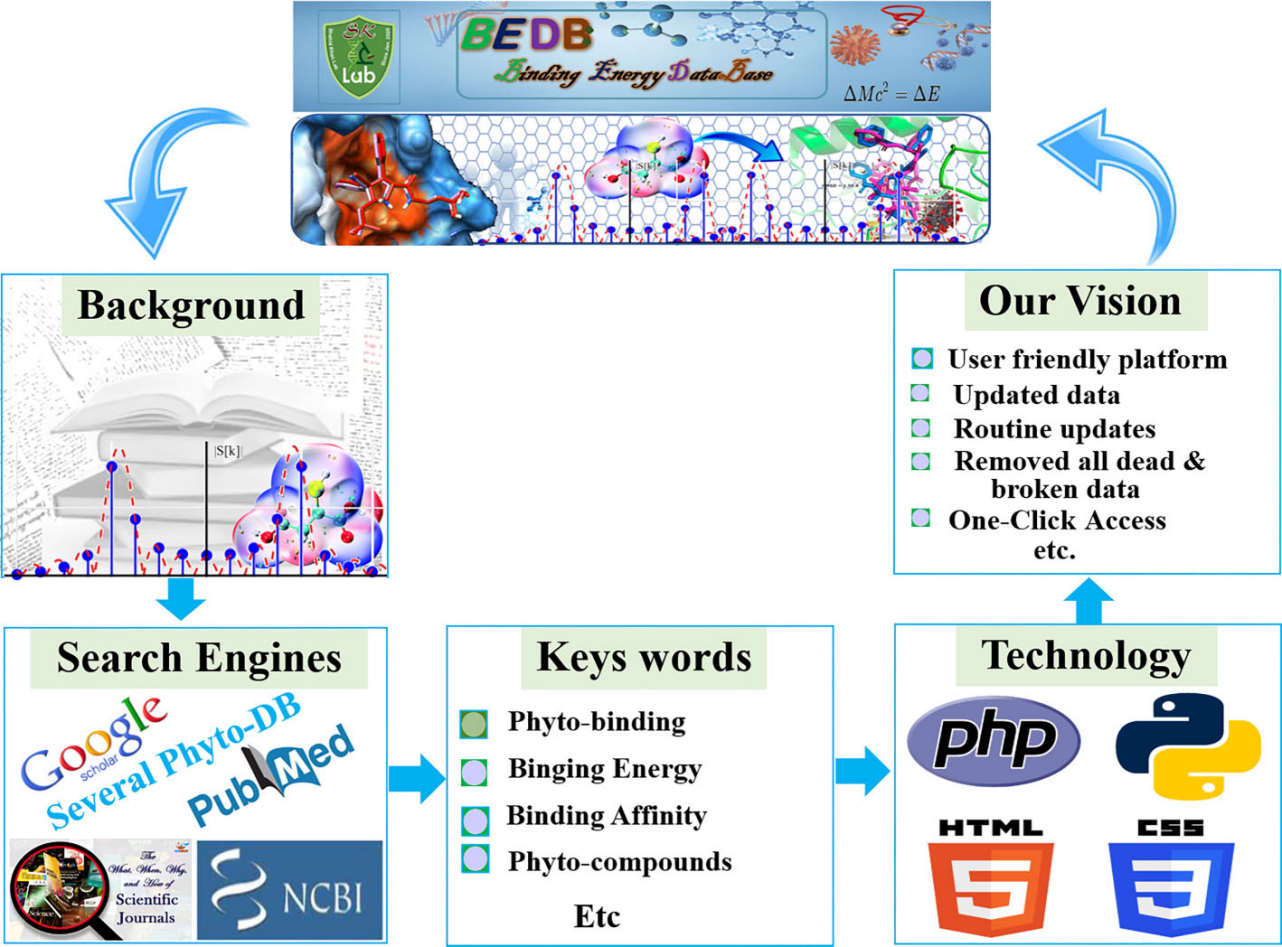
A graphical representation of the BEDB illustrates several key components. It provides a background understanding that offers context for the database. The section on data search engines highlights the various search engines used for data collection. Additionally, the specific keywords utilized during data gathering are listed, and the programming techniques employed in the database creation are outlined. Finally, the objectives and vision of the database are detailed, showcasing the goals and aspirations that underpin its development.

We integrated data from multiple sources, including Google, Google Scholar [21], PubMed [22], and other public databases. We used various keywords such as ‘Binding energy,’ ‘Compound binding energy,’ ‘molecular docking of Compound,’ ‘docking score of Compound,’ and databases of molecular docking and MD simulations to retrieve published docking binding energy and related information. The literature database of PubMed (http://www.ncbi.nlm.nih.gov/pubmed) was instrumental in this search. To avoid missing data, we manually collected the latest information from leading-edge research journals such as PDB (https://nph.onlinelibrary.wiley.com/) and BioResource Technology. We ensured the collection of only published, validated data to maintain high quality. The database was constructed using multiple programming languages, including PHP, MySQL, HTML, CSS, and JavaScript. Figure 1 depicts all the steps involved in data collection and database creation, both in graphical representations and the corresponding names. Ultimately, we provided the scientific community with a comprehensive research database that is simple to use and will be updated over time.

### Usage of the BEDB database

**Figure 2:**
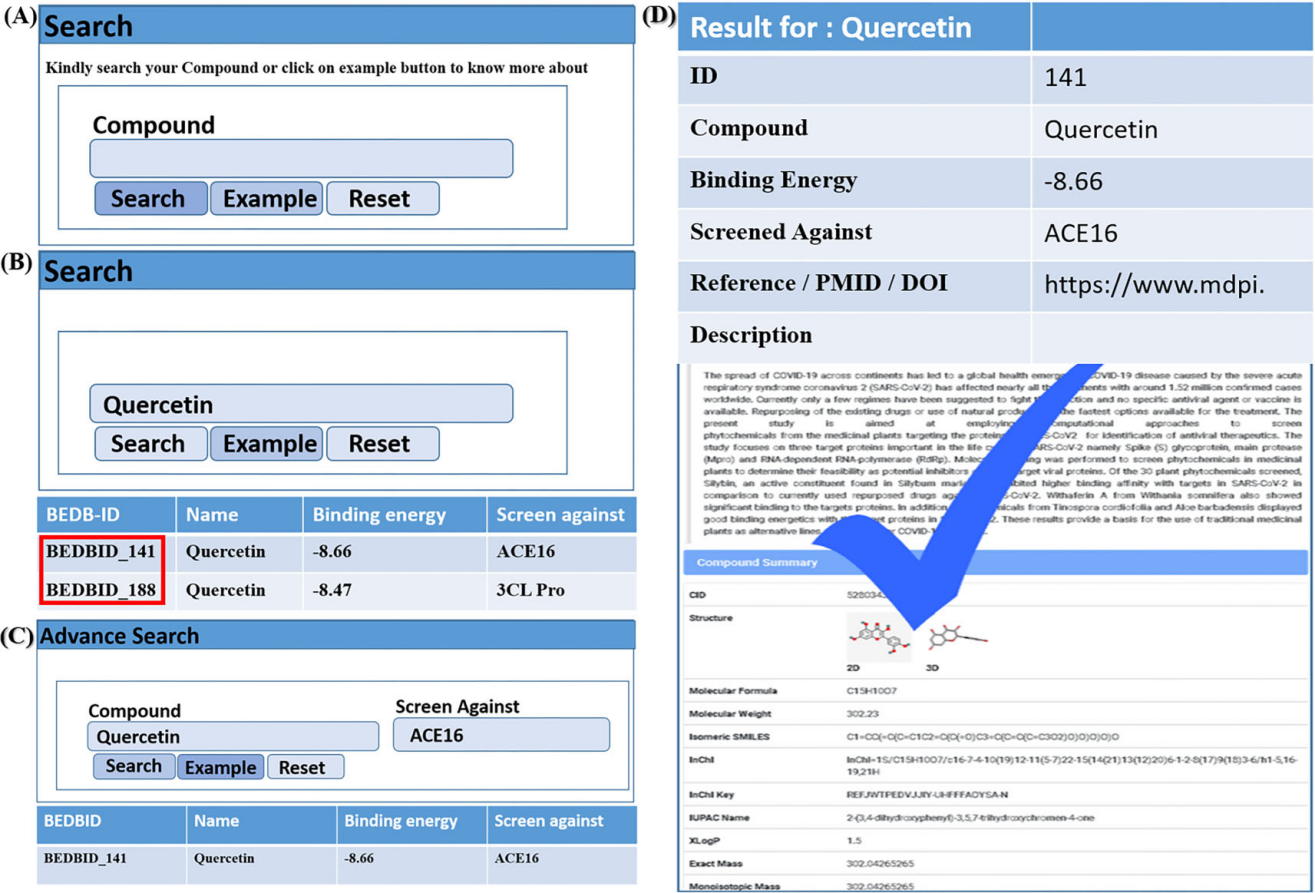
The usage page of the database is organized as: (a) it displays a compound search query, allowing users to search by entering the compound name. (b) By clicking the example button, users can view examples and highlighted BEDB IDs. (c) An advanced search query is also provided, complete with an example. (d) Finally, the detailed results of the searchable compounds are presented.

The BEDB is designed for ease use, offering two search options for quick and efficient data retrieval. Users can search by compound, as illustrated in Fig. 2a. For demonstration purposes, we use ‘quercetin’ as an example (Fig. 2b). Clicking on ‘quercetin’ opens a list showing its binding energy and inhibitors, along with the original BEDB-ID highlighted in red. Further clicking leads to a new window containing comprehensive information about the compound, including the abstract of the published article, authentic references or links, 2D and 3D structures, InChI, InChI Key, molecular formula, molecular weight, isomeric SMILES, and 22 other properties as shown in Fig. 2d. For advanced searches, users can type the compound name and the receptor of interest, or view an example by clicking the example button shown in Fig. 2c. The final search results are displayed in Fig. 2d. Additionally, several options are available on the main database toolbar to enhance usability and authenticity. These include: ‘Usage,’ which details how to use the database. ‘Statistics,’ which provides database statistics. ‘Download,’ which allows users to download all data for scientific research after publishing an article. ‘Useful Links,’ which offers published databases in this research area. Clicking the active button provides access to the database, while the dead button leads to articles about broken databases.

### Molecular docking

The top 10 drugs with the best binding energy were selected for molecular docking and downloaded from PubChem [23] in SDF format. The crystal structure of HMPV PDB ID 8FPJ was downloaded from the protein databank [24]. The HMPV protein is already in complex with MRK-1 inhibitor which we used as a reference drug for molecular docking. Both the protein and the selected drug were minimized utilizing the default parameters in structure minimization and saved in PDB format using UCSF chimera software [25]. Similarly, the 3D structure was prepared with a dock prep tool in Chimera, where charges and polar hydrogen atoms were added, Finally, molecular docking was performed using AutoDock Vina as a plugin in Chimera beneath default parameters on the active residues that bind with MRK-1 inhibitors by using the grid box 60  ×  40  ×  40 Å along with (X, Y, Z) axes, while center at 134.415, 121.893, and 93.9738 Å region [26]. Finally, molecular docking was performed in multiple independent runs for each compound. This approach confirmed the consistency of docking scores and binding interactions, providing further validation of the identified compounds. The top two Compound with the highest binding score was selected for further studies and their two-dimensional interaction was visualized in PyMol.

### MD simulation

To confirm the stability, structural dynamics, conformational changes, and binding interaction between the top 2 compounds, MK-3207 and etoposide, with HMPV virus, MD simulations were performed. We utilized the Amber22 package with ff14SB force field [27,28]. Each protein–ligand complex was solvated in an octahedral water box, maintaining a minimum 1.0 nm distance from the box edge. The simulation box was neutralized by adding Cl^−^ or Na⁺ ions through the Leap module. Energy minimization was performed using both the steepest descent and conjugate gradient algorithms for 6000 and 3000 cycles, respectively, to resolve unfavourable steric clashes [29]. Following gradual heating to 300 K, the system was equilibrated at constant pressure (1 atm), with the temperature controlled by a Langevin thermostat [30]. Subsequently, MD simulations production was conducted for 200 ns on each complex using PMEMD CUDA GTX 1080Ti 11.5 [31]. Long-range electrostatic interactions were computed with the Particle Mesh Ewald method, applying a 10.0 Å cutoff, while covalent bond constraints were managed with the SHAKE algorithm [32]. Trajectory analysis was carried out using the CPPTRAJ module of Amber v22, with data visualization and graphical representation achieved through Origin v2024 and PyMol [33–35].

### Binding free energy calculation

Binding free energy (BFE) calculation is a computational approach frequently used to compute the stability and strength of interaction between a ligand and target protein [36]. It describes the energy variation that occurs when ligands bind to the target protein and form a stable complex under equilibrium conditions. Mathematically, the BFE (Δ*G*_bind_) is defined as the difference between the free energy of the complex (ligand–protein) and the sum of the free energies of the unbound ligand and protein in their respective solvated states. We apply the MMGBSA.PY script to compute the BFE of peptide–protein complexes [37]. The BFE of peptide–protein complexes was calculated by utilizing the last 10 ns MD trajectory taking 500 snapshots based on the following equation:


\begin{eqnarray*}
\Delta G{\mathrm{bind}} = \Delta g{\mathrm{complex}} - [\Delta g{\mathrm{receptor}} + \Delta g{\mathrm{ligand}}]
\end{eqnarray*}

Δ*G*_bind_ represents the total binding energy, while the other components denote the free energy of the complex, receptor, and ligand.

## Results and discussion

### Molecular docking studies

A total of 1321 chemical compound with their binding energy were collected from various published sources and analysed for their potential inhibitory activity against PLpro, ACE, and ECL of the HMPV virus. The highest binding affinities of the top 10 compounds were determined through comprehensive molecular docking analysis, as summarized in Table 1. These compounds exhibited the most favourable binding interactions with the HMPV virus binding pocket, with the corresponding binding affinity values indicating strong potential for antiviral activity. The remaining compounds, which did not exhibit the highest binding affinities, have been systematically incorporated into a comprehensive database for further reference and analysis. This database not only contains the binding affinities of all compounds but also includes additional molecular information. Specifically, for each compound, the database provides critical identifiers and descriptors, including the Compound ID, molecular formula, and both 2D and 3D structural representations. Additionally, the database includes detailed chemical information, such as the Isomeric SMILES, InChI (International Chemical Identifier), and InChI Key, which are essential for precise identification and structural representation. Furthermore, the database contains 22 additional physicochemical properties, providing deeper insight into the compounds’ potential pharmacokinetic and pharmacodynamic profiles. This comprehensive data repository serves as an invaluable resource for further computational studies, aiding in the identification of promising antiviral candidates and facilitating the rational design of novel therapeutic agents. The integration of these detailed molecular descriptors ensures that the database is not only a tool for binding affinity comparison but also a rich source of structural and chemical data for future drug development efforts.

**Table 1:** Docking score of top 10 drugs, their binding energy, as well as interacting residues of HMPV virus.

No	Drugs	PubChem ID	Binding score	Interacting residues
1	Mk3207	25 019 940	−10.3	GLN92, ARG111, TYR58, ILE61, ARG288, LYS307, ARG309, GLU86, LYS87, VAL88, HIS89, GLN193, and THR91
2	Etoposide	36 462	−9.6	HIS1300, ILE1343, GLY1339, PHE1322, GLN1348, and PHE1347
3	Teniposide	452 548	−8.9	GLY1339, ILE1343, HIS1300, PRO973, PHE1311, GLN1348, and PHE1347
4	Uk432097	9 833 519	−8.7	PHE1312, HIS1300, ARG1301, TRP1224, GLN1343, VAL1346, GLN1345, LEU1334, PHE1347, ILE1343, and ASN1340
5	85 019 940	85 019 940	−8.4	VAL1343, PRO973, ARG1307, HIS1300, ARG1301, PHE1347, and ILE1343
6	Setileuton	11 856 170	−8.3	LEU1334, PHE1347, VAL1346, PHE1311, PRO973, and ARG13
7	Orvepitant	9 852 175	−8.1	PHE1347, LEU1334, ILE1343, and ASP1342
8	Cep-11 981	11 751 922	−7.9	VAL1346, PHE1347, ILE1343, LEU1334, ASP1340, ARG1301, and HIS1300
9	Tadalafil	110 635	−7.8	GLN1348, ILE1343, HIS1300, PHE1347, ILE1330, LEU1334, LEU1345
10	Vs-5584	46 912 230	−7.6	ASP1342, PHE1347, and LEU1334

### Development of a new feature

**Figure 3:**
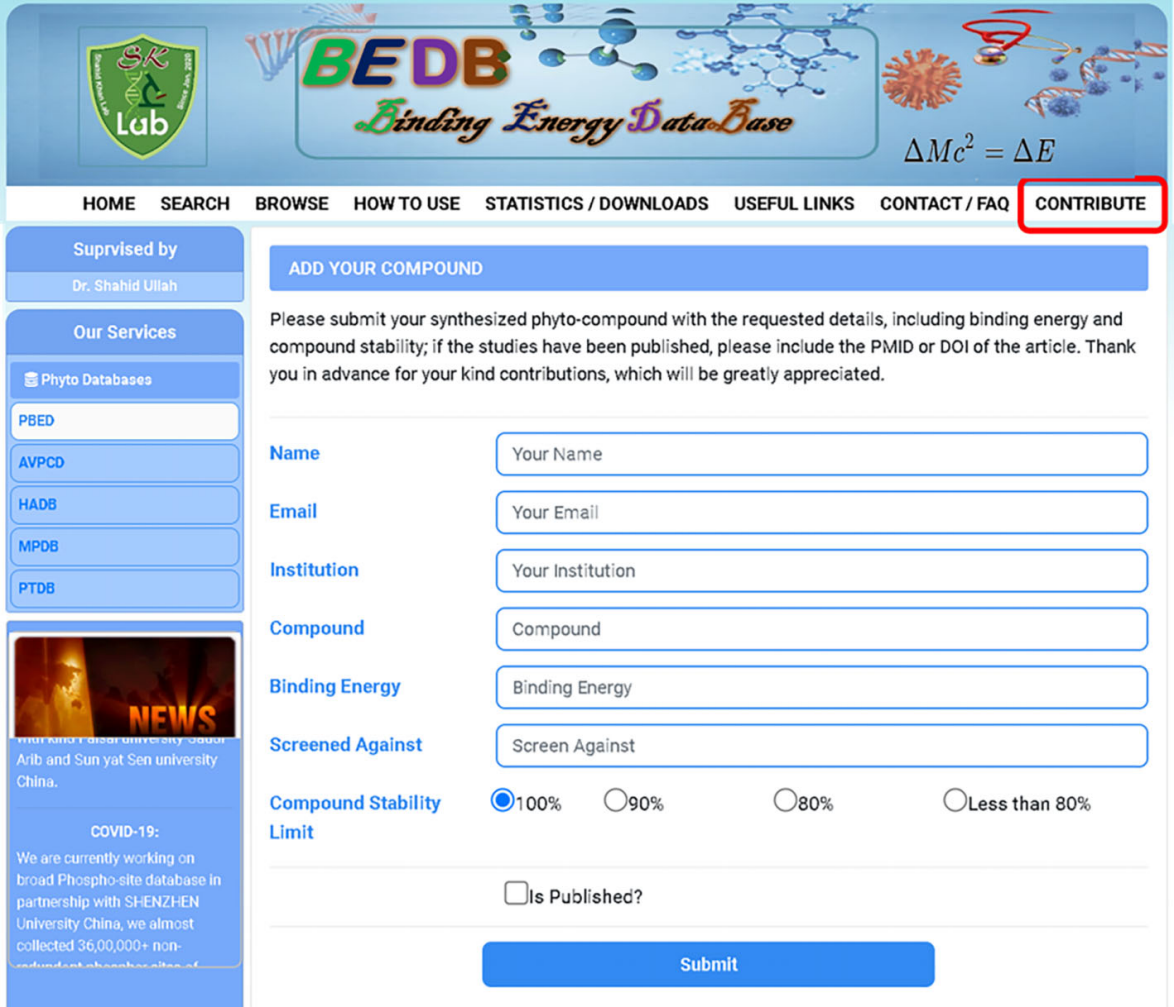
The contribution entry page of the database allows researchers to input new compounds along with their binding energy and other essential information.

To support the scientific community and current docking researchers, we have created a new tool called ‘Contribute,’ located on the main toolbar of the database and marked in red in Fig. 3. This tool allows users to enter new data with relevant information. The goal of this feature is to enable future researchers to obtain the necessary information with a single click, eliminating the need to dock the compound or search through various search engines. Given that the same compound is often docked and published multiple times with the same results in different journals, we aim to save researchers’ time and effort by gathering all relevant information, including binding energy, in one place for molecular docking and MD simulation scientists. The contribution form is depicted in Fig. 3. Furthermore, we provide free access to all researchers across various biological databases and have published multiple databases in renowned worldwide journals. We also encourage all researchers to share and contribute data to relevant databases, which can be accessed through the provided link https://www.pbed.habdsk.org/contribution.

### Molecular docking analysis

**Figure 4:**
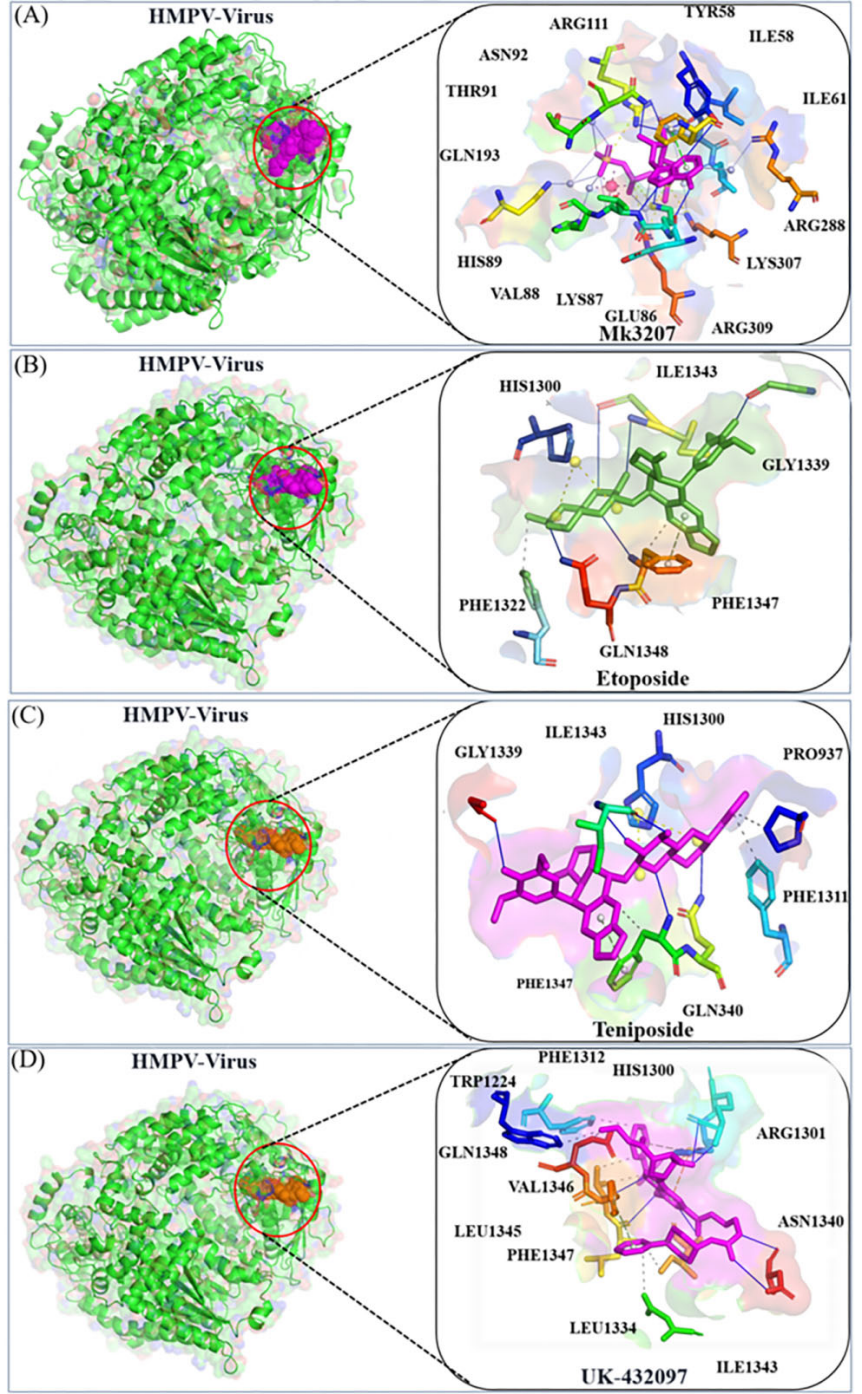
Docking analysis of MK-3207, etoposide, Teniposide, and UK-432097 with the HMPV virus. Panels (a) and (b) show the binding position and 2D interaction of MK-3207 and etoposide, respectively. Panels (c) and (d) depict the binding position and 2D interactions of Teniposide and UK-432097, respectively.

Following the molecular docking procedure, a comprehensive analysis of the binding pockets and the specific residues involved in the interactions was performed. The docking results revealed that MK-3207 exhibited a docking score of −10.7, signifying a strong affinity for the target site. This compound was successfully accommodated within the HMPV virus binding pocket, as shown in Fig. 4. The 2D interaction analysis identified several key residues involved in the binding of MK-3207, including GLN92, ARG111, TYR58, ILE61, ARG288, LYS307, ARG309, GLU86, LYS87, VAL88, HIS89, GLN193, and THR91 (Fig. 4a). These residues play critical roles in mediating the interactions between the ligand and the protein, potentially contributing to the stability and specificity of the binding event. Similarly, etoposide demonstrated a docking score of −9.6, with the 2D interaction analysis revealing a more selective binding profile. This compound interacts primarily with six crucial residues: PHE1322, HIS1300, ILE1343, GLY1339, PHE1347, and GLN1348 (Fig. 4b). These residues are strategically located within the HMPV binding pocket, indicating that etoposide could exert its antiviral effects through specific interactions at these sites. In parallel, Teniposide and UK-432097 also displayed promising docking scores, with Teniposide achieving a score of −8.7 and UK-432097 scoring −8.4. Both molecules fit effectively within the HMPV binding cavity, suggesting their potential as inhibitors of the virus. The interaction analysis for Teniposide highlighted residues such as GLY1339, ILE1343, HIS1300, PRO973, PHE1311, GLN1348, and PHE1347 (Fig. 4c), while UK-432097 was found to engage a broader array of residues, including PHE1312, HIS1300, ARG1301, TRP1224, GLN1343, VAL1346, GLN1345, LEU1334, PHE1347, ILE1343, and ASN1340 (Fig. 4d). The involvement of these multiple residues suggests that UK-432097 might induce a more complex binding interaction compared to the other compounds, potentially leading to a more robust antiviral effect.

These docking analyses underscore the ability of MK-3207, etoposide, Teniposide, and UK-432097 to effectively bind to the HMPV virus binding pocket, with each compound interacting with a distinct set of residues. The variety of interactions observed across the different compound’s points to the specificity and potential therapeutic implications of these molecules in the context of HMPV inhibition. These findings provide valuable insights into the structural basis of drug binding and offer a foundation for further experimental validation and development of targeted antiviral therapies against HMPV.

### Two-dimensional analysis

**Figure 5:**
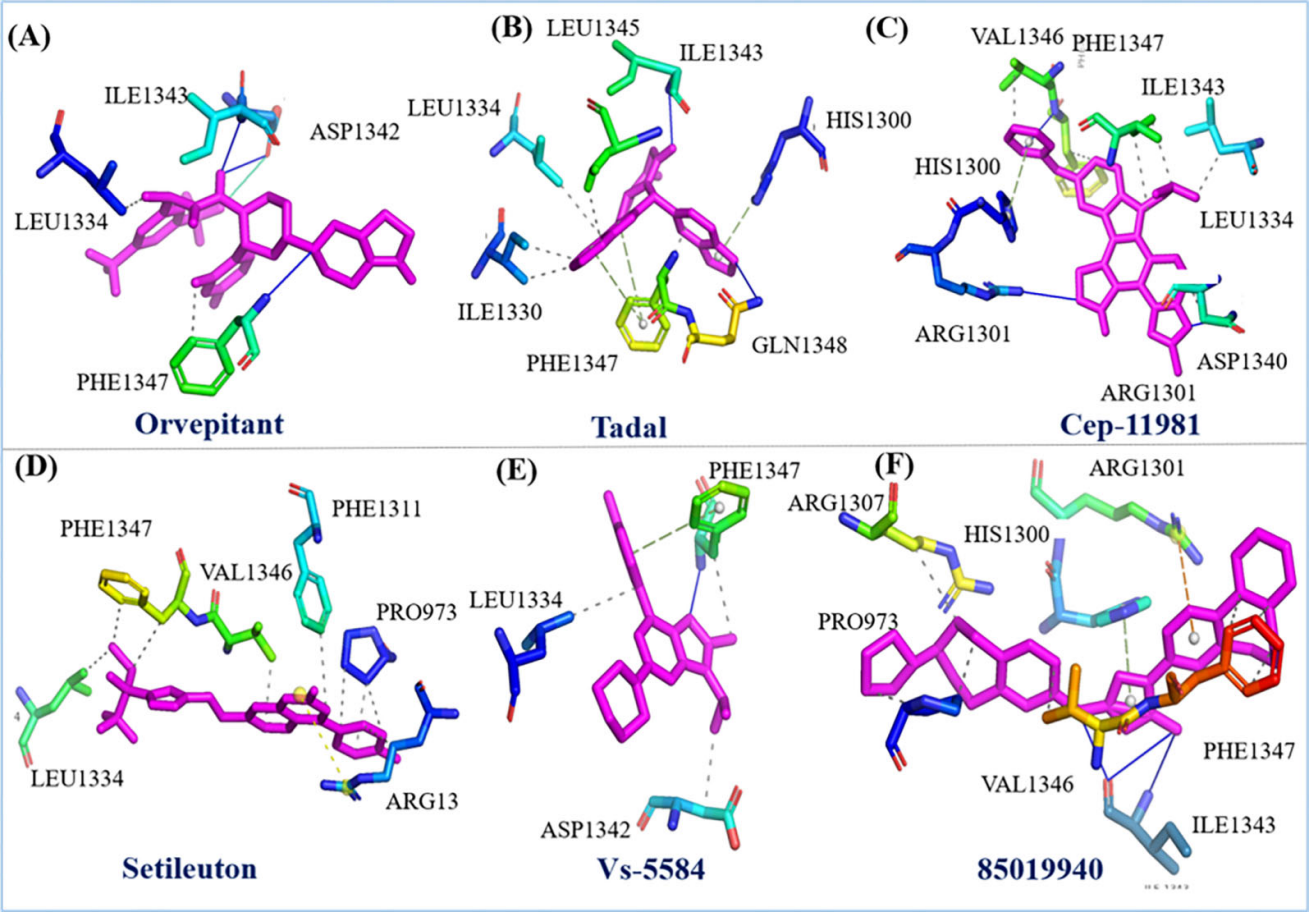
Represent the two-dimensional interaction of six drugs their binding pose and interacting residue with HMPV virus.

In addition to the previously discussed molecular docking analysis, we have extended our investigation by visualizing the interactions of six different drugs with the HMPV virus binding pocket. The detailed 2D interaction diagrams, along with the corresponding binding scores, are presented in Fig. 5 and Table 1, which provide a clearer understanding of the molecular interactions at play. Specifically, the interaction profiles of Orvepitant, Tadalafil, and Cep-1191 reveal a variety of crucial binding events involving specific residues. In the case of Orvepitant, residues ILE1343 and ASP1342 form hydrogen bonds, contributing to the stability of the ligand binding (Fig. 5a). Additionally, LEU1334 and PHE1343 are involved in other types of interactions, such as hydrophobic contacts or van der Waals forces. For Tadalafil, the interaction analysis identifies GLN1348 and ILE1343 as forming a hydrogen bond, while other residues such as HIS1300, PHE1347, ILE1330, LEU1334, and LEU1345 contribute to various non-covalent interactions (Fig. 5b). Similarly, Cep-1191 interacts with ARG1301 through a hydrogen bond, while HIS1300, VAL1346, PHE1347, ILE1343, LEU1334, and ASP1340 engage in additional binding interactions (Fig. 5c). These results suggest a complex and multifaceted binding mechanism for each of these compounds, highlighting their potential for inhibiting HMPV through a variety of interaction modes.

Furthermore, we extended the analysis to include Setileuton, VS-5584, and 85 019 940, whose docking interactions with the HMPV binding pocket were also thoroughly investigated. Setileuton was found to interact with the residues LEU1334, PHE1347, VAL1346, PHE1311, PRO973, and ARG13 (Fig. 5d), with LEU1334, PHE1347, and VAL1346 forming the core of the binding interaction, while additional residues such as PHE1311 and PRO973 contribute to stabilizing the interaction. VS-5584 exhibited interactions primarily with ASP1342, PHE1347, and LEU1334 (Fig. 5e), where PHE1347 and LEU1334 are involved in hydrophobic contacts, and ASP1342 likely plays a role in ionic or hydrogen bonding. 85019940 demonstrated a binding profile where PHE1347, ILE1343, and VAL1343 participated in hydrogen bonding, while PRO973, ARG1307, HIS1300, ARG1301 contributed to additional binding interactions (Fig. 5f). These data indicate a strong affinity of these compounds for the HMPV binding pocket, with specific residues mediating essential interactions that could contribute to their antiviral activity. In a comparative analysis, the protein of interest was subjected to inhibition by flavin-adenine dinucleotide (FAD), a clinically utilized compound known to inhibit HMPV [38]. FAD was included as a benchmark to assess the effectiveness and binding characteristics of the new compounds in comparison. The binding affinity and interaction profiles of the investigated drugs were compared against those of FAD, offering valuable insights into their potential therapeutic efficacy. These results provide a comprehensive understanding of the molecular interactions between the drugs and the HMPV binding pocket, which could serve as a foundation for further experimental validation and the development of novel antiviral therapies targeting HMPV. The variation in interaction patterns among these compounds underscores the complexity of designing effective inhibitors and highlights the need for a multifaceted approach to drug discovery in the fight against viral infections.

### RMSD and RMSF analysis

**Figure 6:**
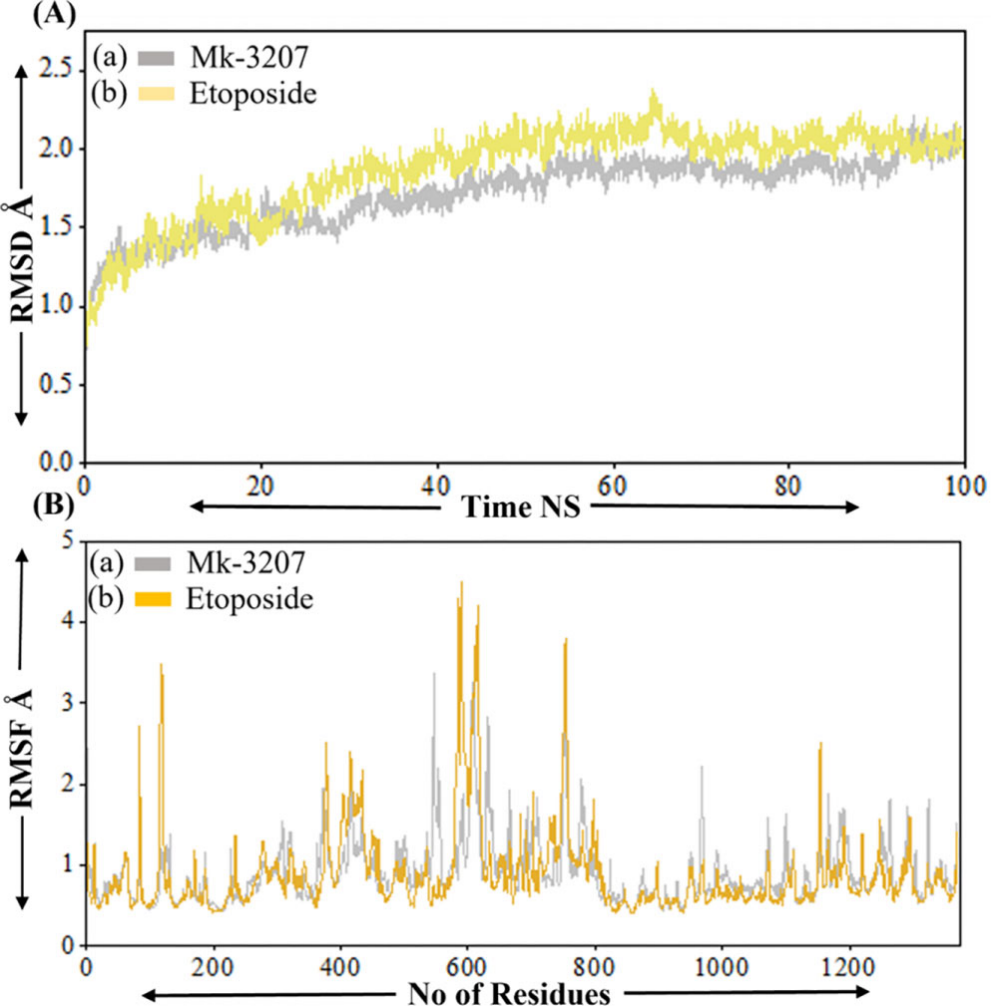
Represent the RMSD and RMSF analysis of MK-3207 and etoposide drugs in complex with HMPV virus. Panel [a(a)] represents the RMSD of MK-3207, panel [a(b)] represents the RMSD of etoposide, panel [b(a)] represents the RMSF of MK-3207, and panel [b(b)] represents the RMSF of etoposide.

The root mean square deviation (RMSD) is an essential parameter in MD simulation, offering insight into the structural stability and conformational changes of molecules, particularly proteins or peptides over time [39]. We performed RMSD analysis of the top two compounds MK-3207 and etoposide in complex with HMPV virus, revealing a significant understanding of their conformational stability and structural dynamics over 100 ns MD simulation. The RMSD profile of MK-3207 initially determines the gradual increase reaching ∼2.0 Å and stabilizing around this value after 60 ns as shown in Fig. 6a(a). On the other hand, the etoposide displays a relatively stable RMSD profile with smaller fluctuations during the simulation period, which preserves its structural integrity and does not experience significant conformational deviations throughout the simulation as shown in Fig. 6a(b) . The root mean square fluctuation (RMSF) is a measure of the average deviation of residue position from their mean positions over time in MD simulation. Root mean square deviation is frequently used in MD simulations to investigate the flexibility of different regions inside biomolecular complexes [40]. The RMSF analysis of MK-3207 and etoposide provides a comprehensive understanding of the stability and flexibility of the molecular system. The RMSF profile of MK-3207 and etoposide in complex with HMPV virus demonstrates generally lower RMSF value among most residues which indicates a more rigid and stable with smaller variability in atomic position as demonstrated in Fig. 6b(a and b). Some peaks in the RMSF profile of MK-3207 are predominantly remarkable as they highlight residues that may perform critical roles in the MDs of the compound, potentially influencing its binding affinity and overall interaction profiles with target proteins. The RMSD and RMSF analysis of these two compounds confirms the inhibitory activity against the HMPV virus.

### SASA and radius of gyration analysis

**Figure 7:**
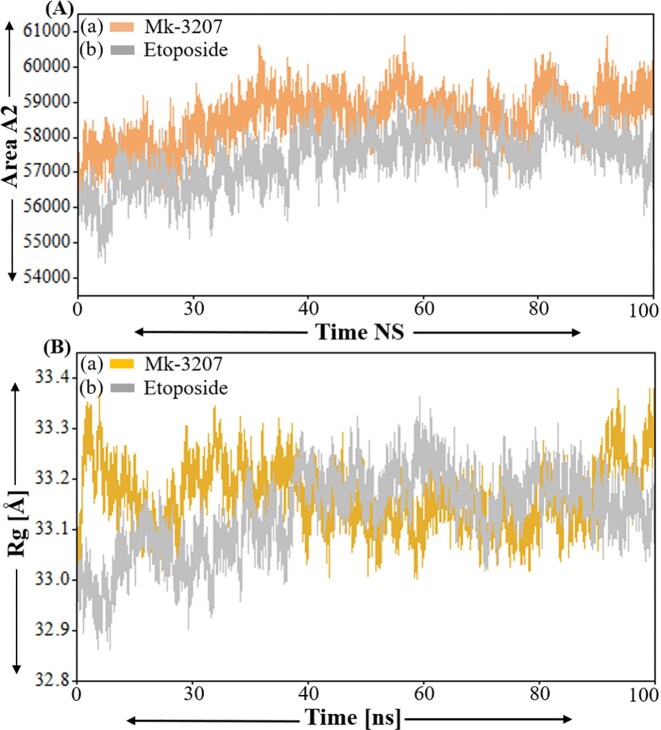
Represent the SASA and radius of gyration of MK-3207 and etoposide in complex with HMPV virus. Panel [a(a)] represents the SASA value of MK-3207, panel [a(b)] represents the SASA value of etoposide. While panel [b(a and b)] represent the Rg of MK-3207 and etoposide, respectively.

Solvent accessible surface area (SASA) represents the surface area of biomolecules accessible to solvent, usually water during MD simulation [41]. This analysis is essential for understanding molecular interaction, folding, and dynamic folding which provide insights into the exposure of hydrophobic and hydrophilic regions of a molecule [42]. SASA also offers insights into ligand binding, protein–solvent interaction a thermodynamic characteristic of biomolecular systems [43]. The SASA values for MK-3207 display a gradual increase, stabilizing around 60 000 Å², demonstrating the development of the molecular surface area as shown in Fig. 7a(a). In contrast, etoposide demonstrates a more stable SASA profile fluctuating around 55 000 Å², which reveals that its surface accessibility remains relatively constant during the whole simulation period as shown in Fig. 7a(b). This stability in SASA values of MK-3207 and etoposide suggests a rigid molecular structure and does not change its exposure to the solvent. The radius of gyration is a crucial parameter in MDs simulation, indicating the special distribution of atoms in a protein or peptide relative to its center of mass. ROG is widely used to examine the overall compactness and folding behaviour of macromolecules during simulation. The RG value of MK-3207 revealed an increasing trend ranging from ∼33.0 to 34.0 Å indicating a gradual expansion of molecular conformation throughout the simulation and this expansion may correlate with the previously observed increase in SASA. On the other hand, etoposide shows a relatively stable RG value fluctuating around 33.5 Å demonstrating that its structural compactness is maintained throughout the simulation as shown in Fig. 7b(a and b). The stability in etoposide Rg shows its conformation remains constant supporting its characterization as a more rigid structure.

### Hydrogen bond analysis

**Figure 8:**
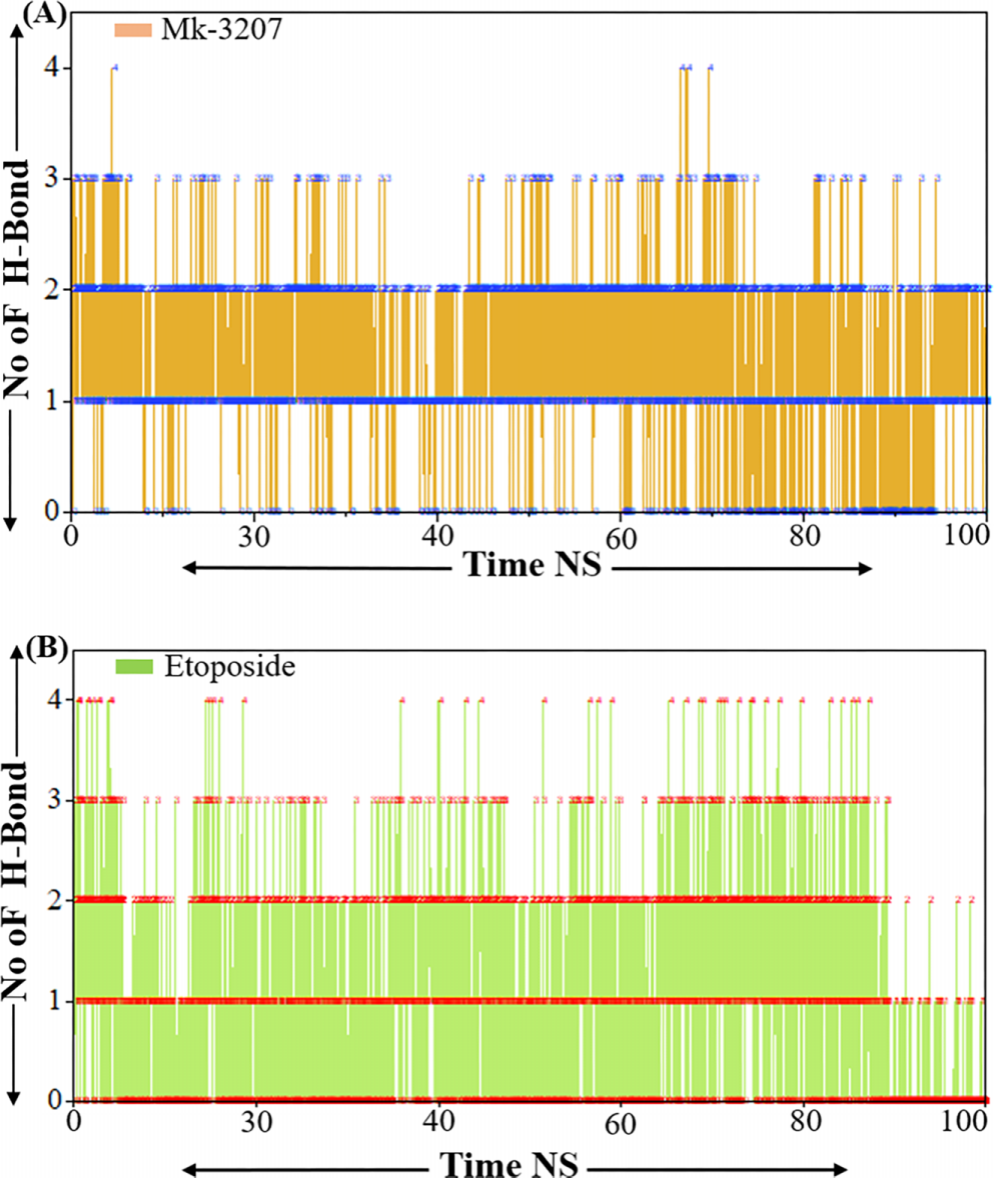
Representations the hydrogen bond analysis of MK-3207 and etoposide. Panel (a) represents the H-bond analysis of MK-3207 and panel (b) represents the H-bond analysis of etoposide.

Hydrogen bonds are essential for maintaining the structure stability and conformation of ligands and proteins as they play a crucial role in secondary and tertiary structure elements. The development of intermolecular hydrogen bonds (H-bonds) significantly affects the stability and orientation of a protein–peptide complex [29]. Investigating these H-bonds can help evaluate a ligand binding strength within a protein’s binding site. The number of hydrogen bonds formed by MK-3207 through the simulation was visualized in the figure. The analysis revealed that MK-3207 maintains a relatively stable number of hydrogen bonds during the simulations as shown in Fig. 8a. The consistency in hydrogen bond interaction revealed a strong network of interactions that may contribute to the stability and potential biological activity of MK-3207. The peaks observed in the number of hydrogen bonds can be correlated with specific conformational states or interactions with water molecules, underscoring the adaptive nature of this compound in a biological environment. While etoposide shows a higher average number of hydrogen bonds during the simulation demonstrating a stronger hydrogen bonding network as shown in Fig. 8b. The fluctuations in etoposide’s hydrogen bond suggest that etoposide forms a stable set of interactions that may enhance its structural integrity and solubility. This stronger hydrogen bonding capacity may contribute to etoposide’s pharmacological effectiveness by facilitating interactions with biological targets. Finally, the hydrogen bond analysis confirms the inhibitory activity and validates the previous results.

## Discussion

Biological databases are essential for comprehensively understanding bioactive compounds and their interactions within living systems. These databases collect and present critical information about chemical structures, properties, biosynthesis, and biological activities, enabling researchers to explore therapeutic potentials and ecological roles. The integration of this data into computational models is foundational for accelerating drug discovery, advancing personalized medicine, and fostering sustainable agricultural practices.

In developing the HABDSK platform, which integrates 22 biological databases [44], we aim to illustrate its utility in the context of drug development. Our molecular docking studies against HMPV identified promising compounds such as MK-3207 and etoposide, which demonstrated strong binding affinities. This highlights BEDB’s role in facilitating the identification of potential antiviral agents and supporting targeted therapeutic development.

To enhance collaboration within the scientific community, we introduced the ‘Contribute’ tool, allowing researchers to add new data efficiently. This feature gathers relevant information, significantly reducing redundancy and streamlining access to vital data for molecular docking and dynamics simulations. Moreover, by making these resources freely accessible, we encourage ongoing contributions, fostering a collaborative atmosphere for future research.

In an era where research methodologies are rapidly evolving, the need for updated, user-friendly biological databases remain pressing. Our platform not only provides a wealth of information but is also designed to enhance reproducibility and transparency, making it a crucial resource for researchers in the life sciences.

## Conclusion

The establishment of our comprehensive compound binding energy database marks an important advancement for researchers in molecular docking and drug discovery. By providing free access to binding energies, essential compound data, and docking results, this platform enhances understanding of molecular interactions between compounds and target proteins while securely storing docking and screening data for future use. The successful docking of the top 10 compounds against the HMPV virus protein demonstrates the significant therapeutic potential of these compounds in combating viral diseases. Our analysis reveals critical binding residues and interactions with the viral protease, which may inform the design of more effective treatments.

Additionally, the user-friendly interface of the database promotes accessibility, encouraging collaboration across the scientific community. This initiative underscores the potential of chemical compounds as viable alternatives in traditional drug development, paving the way for future studies in molecular docking and antiviral therapies. The insights gleaned from our research not only contribute to identifying novel drug candidates but also support the advancement of personalized medicine. As biological databases continue to play a crucial role in life sciences research, our work addresses the need for updated platforms in molecular docking and dynamics, fostering innovation and progress in the field.

## Data Availability

All relevant data are included in the paper can be downloaded directly from the database using the provided link. Additionally, the data will be available according to the journal’s rules and regulations.
